# Family Perceptions of Palliative Care Consultations for Nursing Home Residents

**DOI:** 10.1093/geroni/igaf122.4243

**Published:** 2025-12-31

**Authors:** Alfred Boakye, Kathleen Unroe, Mary Ersek, Peiyuan Zhang, Gretchen Tucker, Alex Floyd, John Cagle

**Affiliations:** University of Maryland, Baltimore, Baltimore, Maryland, United States; Indiana University School of Medicine, Indianapolis, Indiana, United States; University of Pennsylvania, Philadelphia, Pennsylvania, United States; University of Alabama, Tuscaloosa, Alabama, United States; University of Maryland, Baltimore School of Medicine, Baltimore, Maryland, United States; Indiana University School of Medicine, Indianapolis, Indiana, United States; University of Maryland, Batimore, Maryland, United States

## Abstract

People who receive care in nursing homes have a significant burden of serious medical illness, functional impairment, and cognitive impairment, and are known to have unmet palliative care needs. External specialist providers often conduct palliative care consultations (PCCs). PCCs usually include comprehensive physical and psychosocial assessments with accompanying treatment recommendations, goals of care conversations, and support for family members. However, the current understanding of specific interactions that can help improve the quality of PCC visits is limited. This makes the involvement and perceptions of families, who are key stakeholders in care decision-making, critical. Using a descriptive qualitative research design, our study sought to bridge this knowledge gap. As part of an NIH-funded clinical trial (R01 AG066922), we conducted 22 semi-structured interviews with family members of residents with moderate-to-severe cognitive impairment who received PCCs. Residents resided in 1 of 5 nursing homes in either Maryland or Indiana. Family members were recruited within 1 month of the occurrence of a PCC for their associated resident. Data were analyzed using thematic content analysis. According to family members, PCCs promoted physical comfort and family support, attended to residents’ social needs, and provided next steps/care planning. Family also reported PCCs were helpful for reasons including that they improved residents’ quality of life through offering recommendations/referrals, keeping family informed, and supporting family. Findings provide insights and demonstrate the value of PCCs for family members of residents with dementia.

